# Rikkunshito for Preventing Chemotherapy-Induced Nausea and Vomiting in Lung Cancer Patients: Results from 2 Prospective, Randomized Phase 2 Trials

**DOI:** 10.3389/fphar.2017.00972

**Published:** 2018-01-16

**Authors:** Toshiyuki Harada, Toraji Amano, Tomoo Ikari, Kei Takamura, Takahiro Ogi, Toshiaki Fujikane, Yuka Fujita, Kageaki Taima, Hisashi Tanaka, Takaaki Sasaki, Shunsuke Okumura, Shunichi Sugawara, Hiroshi Yokouchi, Noriyuki Yamada, Naoto Morikawa, Hirotoshi Dosaka-Akita, Hiroshi Isobe, Masaharu Nishimura

**Affiliations:** ^1^Center for Respiratory Diseases, JCHO Hokkaido Hospital, Sapporo, Japan; ^2^Clinical Research and Medical Innovation Center, Hokkaido University Graduate School of Medicine, Sapporo, Japan; ^3^Department of Respiratory Medicine, Obihiro-Kosei General Hospital, Obihiro, Japan; ^4^Department of Respiratory Medicine, National Hospital Organization Asahikawa Medical Center, Asahikawa, Japan; ^5^Department of Respiratory Medicine, Hirosaki University Graduate School of Medicine, Hirosaki, Japan; ^6^Respiratory Center, Asahikawa Medical University Hospital, Asahikawa, Japan; ^7^Department of Pulmonary Medicine, Sendai Kousei Hospital, Sendai, Japan; ^8^Department of Pulmonary Medicine, Fukushima Medical University, Fukushima, Japan; ^9^Department of Respiratory Medicine, Iwamizawa Municipal General Hospital, Iwamizawa, Japan; ^10^Division of Pulmonary Medicine, Allergy, and Rheumatology, Department of Internal Medicine, Iwate Medical University, Morioka, Japan; ^11^Department of Medical Oncology, Hokkaido University Graduate School of Medicine, Sapporo, Japan; ^12^Respiratory Center, KKR Sapporo Medical Center, Sapporo, Japan; ^13^First Department of Medicine, Hokkaido University Hospital, Sapporo, Japan

**Keywords:** herbal medicine, nausea, vomiting, lung cancer, chemotherapy

## Abstract

The herbal medicine rikkunshito has the potential to improve chemotherapy-induced nausea and vomiting (CINV) by stimulating ghrelin secretion. We aimed to evaluate the efficacy and safety of rikkunshito in preventing CINV for patients with lung cancer. Two separate prospective, randomized, phase II parallel design studies were conducted in patients with lung cancer. Fifty-eight and sixty-two patients scheduled to receive highly emetogenic chemotherapy (HEC) and moderately emetogenic chemotherapy (MEC), respectively, were randomized 1:1 to receive either standard antiemetic therapy in accordance with international guidelines (S group) or standard antiemetic therapy plus oral rikkunshito (R group). The primary endpoint was overall complete response (CR)—that is, no emesis and rescue medication in the first 120 h post-chemotherapy. Secondary endpoints included CR in the acute (0–24 h) and delayed (>24–120 h) phases and safety. Fifty-seven patients (S group, 28; R group, 29) receiving HEC and sixty-two patients (S group, 30; R group, 32) receiving MEC with comparable characteristics were evaluated. The CR rates were similar across the S and R groups for the HEC study in the overall (67.9% vs. 62.1%), acute (96.4% vs. 89.6%), and delayed (67.9% vs. 62.1%) phases, respectively, and for the MEC study in the overall (83.3% vs. 84.4%), acute (100% vs. 100%), and delayed (83.3% vs. 84.4%) phases, respectively. No severe adverse events were observed. Although rikkunshito was well tolerated, it did not demonstrate an additional preventative effect against CINV in lung cancer patients receiving HEC or MEC.

**Clinical Trial Registry Information:** This study is registered with the University Hospital Medical Information Network (UMIN) Clinical Trial Registry^1^, identification numbers UMIN 000014239 and UMIN 000014240.

## Introduction

Some of the most prevalent and concerning effects of cancer treatment are chemotherapy-induced nausea and vomiting (CINV) (Hofman et al., 2004; Molassiotis et al., 2008). CINV leads to reduced chemotherapy adherence rates, deteriorated of function and quality of life (QOL), and aggravated anxiety and depression (Schwartzberg, 2007; Hesketh, 2008). Therefore, circumvention of CINV is a critical element of supportive care in cancer.

In recent years, the incidence of CINV has been decreasing through the improvement of antiemetic agents (Warr et al., 2005; Botrel et al., 2011; Hesketh et al., 2014) and refinements of antiemetic guidelines by the American Society of Clinical Oncology (ASCO) (Hesketh et al., 2016), Multinational Association of Supportive Care in Cancer/European Society of Medical Oncology (MASCC/ESMO) (Jordan et al., 2011), National Comprehensive Cancer Network (NCCN) (National Comprehensive Cancer Network [NCCN], 2016), and Japan Society of Clinical Oncology (JSCO) (Japan Society of Clinical Oncology [JSCO], 2015). However, CINV still occurs in approximately half of patients who receive chemotherapy for cancer (Aapro et al., 2012), and additional CINV prevention methods are required.

The emetogenicity of anti-cancer agents has been categorized according to their risk levels in the guidelines set by ASCO (Hesketh et al., 2016), MASCC/ESMO (Jordan et al., 2011), NCCN (National Comprehensive Cancer Network [NCCN], 2016), and JSCO (Japan Society of Clinical Oncology [JSCO], 2015). A chemotherapy regimen that is associated with emesis in ≥90% of patients is considered to have high emetic risk (highly emetogenic chemotherapy, HEC), regimens that cause emesis in 30–90% of patients are considered to have a moderate emetic risk (moderately emetogenic chemotherapy, MEC), those causing emesis in 10–30% of patients have a low emetic risk, and those causing emesis in less than 10% of patients have a minimum emetic risk. In all clinical guidelines, cisplatin (CDDP) is classified as HEC and carboplatin (CBDCA) as MEC. With these regimens, the patients are at risk of developing CINV for up to 120 h after receiving chemotherapy; this 120-h watch period for CINV incorporates an acute phase (0–24 h), delayed phase (>24–120 h), and overall phase (0–120 h).

The treatments recommended by the international antiemetic guidelines of ASCO (Hesketh et al., 2016), MASCC/ESMO (Jordan et al., 2011), NCCN (National Comprehensive Cancer Network [NCCN], 2016), and JSCO (Japan Society of Clinical Oncology [JSCO], 2015) for the prevention of CINV that is associated with HEC are a neurokinin 1 receptor antagonist (NK-1-RA), a serotonin receptor antagonist (5HT_3_-RA), and dexamethasone (DEX); those for MEC include 5HT_3_-RA, DEX, and (optionally) NK-1-RA.

Herbal medicines, which were systemically popularized in Japan in the 16th century, have a wide range of indications aimed at maintaining QOL in patients rather than curing them (Mizukami et al., 2009). Herbal medicines are thus intended to boost the body’s own healing power (i.e., immune system) and help restore its natural balance. Rikkunshito, an herbal medicine, has been shown to improve upper gastrointestinal symptoms and anorexia (Tomono et al., 2006; Ohno et al., 2011; Arai et al., 2012; Tominaga et al., 2012); therefore, we hypothesized that this herbal medicine can reduce CINV. Rikkunshito was approved in Japan only as a fixed dose of 7.5 g (2.5 g three times a day).

To the best of our knowledge, there are no prospective studies on the efficacy of herbal medicines in preventing CINV. Herein, we describe the results of two separate prospective, randomized, phase II parallel design studies that evaluated the efficacy and safety of rikkunshito in the prevention of CINV in patients with lung cancer receiving CDDP-based HEC (HOT1402) and CBDCA-based MEC (HOT1403).

## Materials and Methods

This study comprised two separate prospective, randomized phase II Hokkaido Lung Cancer Study Group Trial (HOT) investigations that were conducted in accordance with the principles of the Declaration of Helsinki, Good Clinical Practice guidelines (World Medical Association, 1997), and CONSORT guidelines. The protocol was approved by the institutional review boards of all participating institutions, and all patients provided written informed consent before treatment. This study was registered at the University Hospital Medical Information Network (UMIN) Clinical Trials Registry as UMIN000014239 (HOT1402) and UMIN000014240 (HOT1403).

### Patient Eligibility

Eligible patients met the following criteria: histologic or cytologic confirmation of lung cancer; age ≥20 years; an Eastern Cooperative Oncology Group (ECOG) performance status (PS) score of 0–2; treatment with CDDP-based HEC (HOT1402) or CBDCA-based MEC (HOT1403); adequate bone marrow function (leukocyte count ≥3,000/mm^3^, neutrophil count ≥1,500/mm^3^, platelet count ≥100,000/mm^3^, and hemoglobin content ≥9.0 g/dL); adequate function in other organs (total bilirubin concentration ≤1.5 mg/dL, aspartate transaminase and alanine transaminase levels ≤100 IU/L, and creatinine clearance ≥60 mL/min [HEC] or ≥50 mL/min [MEC]); P_a_O_2_ ≥60 Torr, or S_p_O_2_ ≥92%; and a life expectancy of 2 months or more. Patients who previously used rikkunshito or had active infectious diseases, serious medical complications (e.g., active peptic ulcer, heart disease, diabetes mellitus, cerebrovascular disease, neuropsychiatric disorder), had symptomatic brain metastasis, were lactating or pregnant, or had active concomitant malignancies were ineligible for the study.

### Treatment Plan

Eligible patients were randomized in a 1:1 ratio using a minimization method and were assigned to receive either (1) standard antiemetic therapy in accordance with the ASCO (Hesketh et al., 2016), NCCN (Jordan et al., 2011), MASCC/ESMO (National Comprehensive Cancer Network [NCCN], 2016), or JSCO (Japan Society of Clinical Oncology [JSCO], 2015) guidelines at the investigators’ discretion (the S group) or (2) standard antiemetic therapy plus 2.5 g of oral rikkunshito three times a day on days 1–7 (the R group). The stratification factors included sex, habitual alcohol intake (yes or no), and palonosetron use (yes or no) in the HEC study, and sex, habitual alcohol intake (yes or no), and NK-1-RA use (yes or no) in the MEC study.

### Assessment

The efficacy and safety of the antiemetic therapy were evaluated during the 7 days following the administration of the HEC or MEC in the first cycle. The patients recorded episodes of emesis, nausea ratings, and rescue medications taken during the first 120 h, as well as any impairment of eating habits during the first 7 days post-chemotherapy, in a diary. Patients assessed their nausea with a 100-mm horizontal visual analog scale (VAS); scores of ≤5 and ≤25 mm on the VAS scale indicated no nausea or no significant nausea, respectively. The patients also recorded the ratio of dietary intake with a 100-mm horizontal VAS. Adverse events related to the antiemetic treatment were surveyed by the investigators according to the National Cancer Institute Common Terminology Criteria for Adverse Events, version 4.0.

### Objectives

The primary endpoint was the complete response (CR; i.e., no emesis and no rescue medication) rate in the overall post-chemotherapy phase. The secondary endpoints were (1) the CR rate in the acute and delayed phases; (2) complete protection (CP; i.e., no emesis, no significant nausea, and no rescue medication) rate in the acute, delayed, and overall phases; (3) total control (TC; i.e., no emesis, no nausea, and no rescue medication) rate in the acute, delayed, and overall phases; (4) dietary intake during the 7 days post-chemotherapy; and (5) safety.

### Statistical Analysis

These two prospective, randomized phase II studies were designed to assess antiemetic efficacy with regard to the CR rate during the overall phase. The primary endpoint was CR rate in the overall phase among all per-protocol patients. The sample size was determined according to a one-arm binomial design devised by the Southwest Oncology Group. In the HEC study, we estimated the patient accrual number to be 27 assuming that a CR of 80% in eligible patients would indicate potential usefulness while a CR of 55% would be the lower limit of interest (Hesketh et al., 2003; Poli-Bigelli et al., 2003; Longo et al., 2011; Suzuki et al., 2016), with α = 0.05 and β = 0.20. To allow for patient dropouts, we aimed for the enrollment of 58 patients in the HEC study. In the MEC study, the estimated accrual number was 29 patients, assuming that a CR of 75% in eligible patients would indicate potential usefulness while a CR of 50% would be the lower limit of interest (Herrstedt et al., 2009; Aapro et al., 2010; Rapoport et al., 2010), with α = 0.05 and β = 0.20. We aimed to enroll 62 patients in the MEC study to allow for dropouts. Categorical variables were analyzed by using the χ^2^ or Fisher’s exact tests. All *P*-values are 2-sided; a *P*-value of 0.05 indicated statistical significance. Statistical analyses were performed by using Excel 2011 (Microsoft) with the add-in software Statcel 4 (OMS Publishing Inc., Saitama, Japan).

## Results

### Patient Characteristics

**FIGURE 1:**
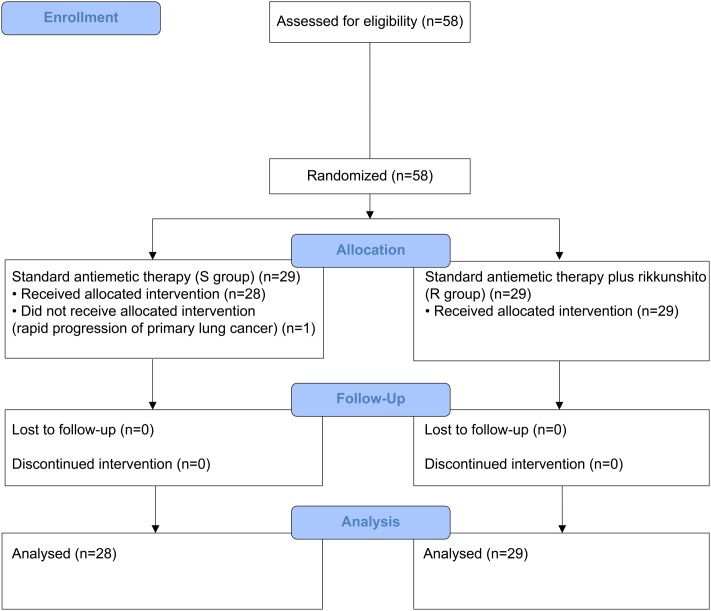
CONSORT diagram showing patients disposition in the highly emetogenic chemotherapy (HEC) study.

**FIGURE 2:**
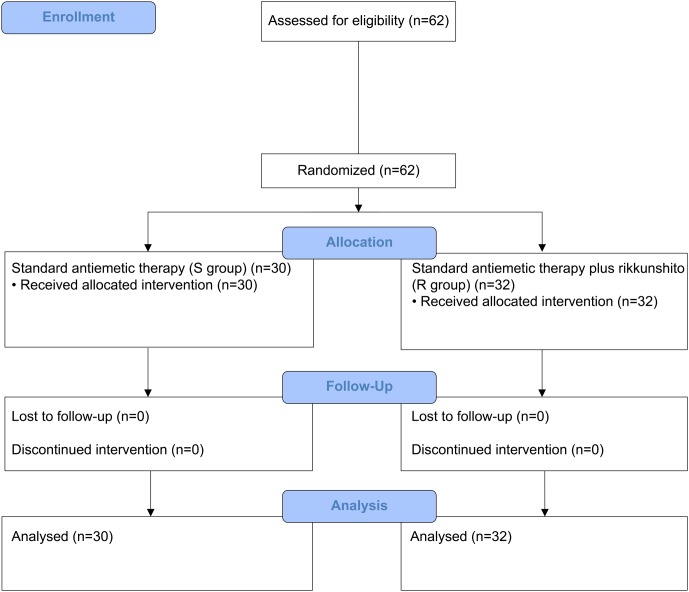
CONSORT diagram showing patients disposition in the moderately emetogenic chemotherapy (MEC) study.

Between July 2014 and January 2016, 58 patients who received HEC (29 each in the S and R groups) and 62 patients who received MEC (30 in the S group and 32 in the R group) were enrolled. One patient from the S group of the HEC study was excluded because of disease progression before the antiemetic treatment; hence, only 57 patients who received HEC were evaluable (**Figures 1**, **2**). All patients enrolled in the two phase II studies had a good nutritional status, and had neither muscle wasting nor weight loss. The patients’ characteristics are summarized in **Table 1**. The baseline characteristics of the study subjects were similar between the groups in both the HEC and MEC studies. In the HEC study, the median patient age was 65 years (range 46–76 years), of whom 77.2% were men and most (96.5%) had a good ECOG PS (0–1). The most common histology was adenocarcinoma (54.4%), followed by small cell carcinoma (24.6%), and squamous cell carcinoma (14.0%). Most of the patients (87.7%) were treated with palliative chemotherapy. The combined chemotherapeutic agents with CDDP were as follows: pemetrexed (29.8%), pemetrexed plus bevacizumab (22.8%), etoposide (14.0%), vinorelbine (8.8%), irinotecan (12.3%), gemcitabine (8.8%), docetaxel (1.8%), and S-1 (1.8%). As for the MEC study, the median patient age was 70 years (range 45–89 years); 77.4% were men and most (95.2%) had a good ECOG PS of 0–1. The most common histology was adenocarcinoma (46.8%), followed by squamous cell carcinoma (30.6%) and small cell carcinoma (12.9%). Most of the patients (95.2%) were treated with palliative chemotherapy. The combined chemotherapeutic agents with carboplatin were as follows: pemetrexed (29.0%), nab-paclitaxel (27.4%), etoposide (17.7%), S-1 (9.7%), paclitaxel plus bevacizumab (6.5%), pemetrexed plus bevacizumab (3.2%), gemcitabine (3.2%), paclitaxel (1.6%), and amrubicin (1.6%).

**Table 1:** Patient characteristics.

	HEC		MEC	
		
	S group (*n* = 28)	R group (*n* = 29)	S group (*n* = 30)	R group (*n* = 32)
Age (years)	65 (46–73)	66 (55–76)	68 (45–79)	71 (49–89)
Sex				
Male	21	23	22	26
Female	7	6	8	6
ECOG performance status				
0	14	17	8	12
1	13	11	20	19
2	1	1	2	1
Histology				
Adenocarcinoma	18	13	13	16
Squamous cell carcinoma	3	5	9	10
Large cell carcinoma	2	0	1	0
Not other specified	0	0	2	0
Small cell carcinoma	5	9	5	3
Large cell neuroendocrine carcinoma	0	1	0	2
Pleomorphic carcinoma	0	1	0	1
Type of chemotherapy				
Adjuvant	5	2	0	3
Palliative	23	27	30	29
Dose of cisplatin (mg/m^2^)				
60	2	6		
75	14	10		
80	12	13		
Dose of carboplatin (AUC)				
4			0	2
5			20	18
6			10	12
Combined drugs with platinum				
Pemetrexed	8	9	9	9
Pemetrexed + Bevacizumab	8	5	1	1
Irinotecan	1	6	0	0
Docetaxel	0	1	0	0
Vinorelbine	5	0	0	0
Gemcitabine	1	4	1	1
S-1	1	0	2	4
Paclitaxel	0	0	1	0
Paclitaxel + Bevacizumab	0	0	2	2
Nab-paclitaxel	0	0	7	10
Amrubicin	0	0	0	1
Etoposide	4	4	7	4


### Efficacy

In the HEC study, the CR rates in the overall phase were 67.9% (95% confidence interval [CI], 47.7–84.1) in S group and 62.1% (95% CI, 42.3–79.3) in R group (*P* = 0.65), which did not meet the primary endpoint. In the MEC study, the CR rates in the overall phase were 83.3% (95% CI, 65.3–94.4) in S group and 84.4% (95% CI, 67.2–94.7) in R group (*P* = 0.59), which met the primary endpoint (**Table 2**). In the HEC and MEC studies, rikkunshito did not exhibit additional improvement on CR rates in the overall phase. Furthermore, rikkunshito did not improve CR rates for the acute and delayed phases in either the HEC or MEC study. Rikkunshito administration also did not improve CP or TC rates in the acute, delayed, or overall phases in the HEC and MEC study. The median dietary intakes were also similar between the S and R groups (89 and 89 mm in the HEC study, and 90 and 91 mm in the MEC study, respectively). Subgroup analyses according to sex, age, alcohol intake, smoking status, ECOG PS, body mass index, motion sickness, dexamethasone dose, treatment line, histology, CDDP, or CBDCA dose, and combined drugs revealed no additional benefit for rikkunshito administration on CR rates in the acute, delayed, or overall phases (data not shown).

**Table 2:** Inter-group comparisons of efficacy outcomes.

	HEC			MEC		
		
	S group (*n* = 28)	R group (*n* = 29)	*P*-value	S group (*n* = 30)	R group (*n* = 32)	*P*-value
No emesis						
Overall	26 (92.9%)	25 (86.2%)	0.41	30 (100%)	28 (90.6%)	0.06
Acute	28 (100%)	28 (96.6%)	0.51	30 (100%)	32 (100%)	1
Delayed	26 (92.9%)	25 (86.2%)	0.41	30 (100%)	28 (90.6%)	0.06
No significant nausea						
Overall	18 (64.3%)	21 (72.4%)	0.51	24 (80.0%)	24 (75.0%)	0.64
Acute	28 (100%)	27 (93.1%)	0.25	29 (96.7%)	30 (93.8%)	0.52
Delayed	18 (64.3%)	21 (72.4%)	0.51	24 (80.0%)	24 (75.0%)	0.64
No nausea						
Overall	16 (57.1%)	16 (55.2%)	0.88	21 (70.0%)	21 (65.6%)	0.71
Acute	26 (92.9%)	26 (89.7%)	0.52	28 (93.3%)	28 (87.5%)	0.37
Delayed	16 (57.1%)	16 (55.2%)	0.88	21 (70.0%)	21 (65.6%)	0.71
Complete response						
Overall	19 (67.9%)	18 (62.1%)	0.65	25 (83.3%)	27 (84.4%)	0.59
Acute	27 (96.4%)	26 (89.7%)	0.32	30 (100%)	32 (100%)	1
Delayed	19 (67.9%)	18 (62.1%)	0.65	25 (83.3%)	27 (84.4%)	0.59
Complete protection						
Overall	17 (60.7%)	18 (62.1%)	0.92	21 (70.0%)	23 (71.9%)	0.87
Acute	27 (96.4%)	26 (89.7%)	0.32	29 (96.7%)	31 (96.9%)	0.73
Delayed	17 (60.7%)	18 (62.1%)	0.92	21 (70.0%)	23 (71.9%)	0.87
Total control						
Overall	15 (53.6%)	15 (51.7%)	0.68	20 (66.7%)	20 (62.5%)	0.73
Acute	26 (92.9%)	25 (86.2%)	0.35	28 (93.3%)	29 (90.6%)	0.53
Delayed	15 (53.6%)	15 (51.7%)	0.89	20 (66.7%)	20 (62.5%)	0.73


### Safety

Rikkunshito was well tolerated, with frequencies of treatment-related adverse events similar to those reported in the S groups. Most adverse events were mild and were associated with the patients’ cancer and/or chemotherapy treatment. The most common treatment-related adverse events were constipation, diarrhea, and hiccups. No severe adverse events attributed to antiemetic treatments were reported in either study (**Table 3**).

**Table 3:** Treatment-related adverse events.

	HEC study				MEC study			
		
	S group	(*n* = 28)	R group	(*n* = 29)	S group	(*n* = 30)	R group	(*n* = 32)
		
	Grade 1/2	Grade 3/4	Grade 1/2	Grade 3/4	Grade 1/2	Grade 3/4	Grade 1/2	Grade 3/4
Constipation	13 (46.4%)	0	13 (44.8%)	0	6 (20.0%)	1 (3.3%)	5 (15.6%)	0
Diarrhea	1 (3.6%)	0	4 (13.8%)	1 (3.4%)	4 (13.3%)	0	1 (3.1%)	0
Hiccups	4 (14.3%)	0	5 (17.2%)	0	0	0	3 (9.4%)	0


## Discussion

To the best of our knowledge, this study was the first prospective trial designed to evaluate the efficacy and safety of herbal medicine for the prevention of CINV in patients with lung cancer receiving HEC and MEC. Herbal medicines are inexpensive dietary supplements that can boost the body’s immune system and have the potential to improve anorexia and CINV in cancer patients. The orexigenic hormone ghrelin is a 28-amino acid peptide and has an *n*-octanoyl modification on Ser3; it was first isolated from rat stomachs and was found to be an endogenous ligand for the receptor of the growth hormone secretagogue. Additionally, ghrelin also has an intense appetite-enhancing effect (Kojima et al., 1999). A decrease in the concentration of circulating ghrelin along with appetite loss has been observed in CDDP-treated rats (Takeda et al., 2008). Administration of exogenous ghrelin peripherally improves anorexia (Liu et al., 2006; Takeda et al., 2008) and vomiting (Rudd et al., 2006) induced by CDDP.

Rikkunshito is an herbal medicine prepared by combining eight herbal medicines: *Atractylodis lanceae rhizoma*, *Ginseng radix*, *Pinelliae tuber*, *Hoelen*, *Zizyphi fructus*, *Aurantii nobilis pericarpium*, *Glycyrrhizae radix*, *and Zingiberis rhizoma* (Suzuki et al., 2009; Mochiki et al., 2010). Rikkunshito stimulates ghrelin secretion from the stomach and the response to it in the hypothalamus (Takeda et al., 2008; Fujitsuka et al., 2009). It is widely used in Japan for the treatment of upper gastrointestinal symptoms in patients with functional dyspepsia (Arai et al., 2012), gastroesophageal reflux disease (Tominaga et al., 2012), and chemotherapy-induced nausea for cancer patients (Tomono et al., 2006; Ohno et al., 2011). Based on these findings, we posited that rikkunshito can improve CINV and conducted this prospective study to evaluate its efficacy for the prevention of CINV in patients with lung cancer who were receiving HEC and MEC.

In the HEC study, the CR rates in the overall phase were 67.9% in the S group and 62.1% in the R group, which did not meet the primary endpoint goals. These results were inferior to previous phase III study results that revealed overall CR rates of 59–77% (Hesketh et al., 2003; Poli-Bigelli et al., 2003; Longo et al., 2011; Suzuki et al., 2016). On the other hand, the overall phase CR rates in our MEC study were 83.3% in the S group and 84.4% in R the group, which met the primary endpoint goals. These results were superior compared to previous phase III trial results that yielded overall CR rates of 54–74% (Herrstedt et al., 2009; Aapro et al., 2010; Rapoport et al., 2010). Despite having the CINV symptoms, patients enrolled in these two studies were diligent with fulfilling the requirements for daily-recommended nutrients. Therefore, the patients had well to excellent food intake throughout the course of these two studies.

In the present study, rikkunshito was safe and manageable; however, it did not demonstrate any additional benefits beyond those of standard antiemetic regimens used for the prevention of CINV in patients receiving HEC and MEC for lung cancer. Moreover, rikkunshito did not show additional benefits when performing subset analyses of various clinical factors. One possibility that remains to be investigated is that rikkunshito did not sufficiently increase the plasma levels of acylated ghrelin (the active form of ghrelin) in the patients included in this study. For ethical reasons, we did not examine the level of ghrelin in each patient.

The frequency of CINV in patients receiving HEC remained high, which is a challenge that remains to be solved. Olanzapine, which inhibits multiple neurotransmitters, has been reported to produce favorable results and could be an attractive treatment option for CINV prevention (Navari et al., 2011; Hashimoto et al., 2016). In the present study, olanzapine was not used as part of the standard antiemetic regimen, but it was used as a rescue medication in one patient who was receiving HEC.

## Conclusion

Rikkunshito was well tolerated; however, it did not show any additional benefits beyond those of standard antiemetic regimens for the prevention of CINV in patients with lung cancer who were receiving HEC and MEC. Further investigation is required to improve CINV control, especially in patients receiving HEC.

## Author Contributions

Conceptualization and design by TH. Data collection by all authors. Data analysis and interpretation done by TH and TA. Manuscript written by TH. Critical review and revisions of manuscript done by all the authors. Finally, all authors have agreed with the content and approve of the manuscript for submission.

## Conflict of Interest Statement

The authors declare that the research was conducted in the absence of any commercial or financial relationships that could be construed as a potential conflict of interest.
